# Medical Management of Dyslipidemia for Secondary Stroke Prevention: Narrative Review

**DOI:** 10.3390/medicina59040776

**Published:** 2023-04-17

**Authors:** Yoonkyung Chang, Soojeong Eom, Minjeong Kim, Tae-Jin Song

**Affiliations:** 1 Department of Neurology, Mokdong Hospital, College of Medicine, Ewha Womans University, Seoul 07985, Republic of Korea; 2 Department of Medicine, College of Medicine, Ewha Womans University, Seoul 07804, Republic of Korea; 3 Department of Neurology, Seoul Hospital, College of Medicine, Ewha Womans University, Seoul 07804, Republic of Korea

**Keywords:** dyslipidemia, stroke, statin, ezetimibe, PCSK9 inhibitor

## Abstract

Dyslipidemia is a major risk factor for stroke, following hypertension, diabetes, and smoking, and is an important risk factor for the prevention and treatment of coronary artery disease and peripheral vascular disease, including stroke. Recent guidelines recommend considering low-density lipoprotein cholesterol (LDL-C)-lowering therapies, such as statins (preferably), ezetimibe, or proprotein convertase subtilisin/kexin type 9 (PCSK9) inhibitors to prevent the occurrence or recurrence of stroke, adhering to the “lower is better” approach. In this review, we examined the evidence supporting lipid-lowering medications like statins, ezetimibe, and PCSK9 inhibitors for secondary stroke prevention and dyslipidemia management in different stroke subtypes. Stroke guidelines advocate for administering the maximum tolerable dose of statins as the primary treatment and as soon as possible despite the potential for new-onset diabetes mellitus and possible muscle and liver toxicity due to their demonstrated benefits in secondary prevention of cardiovascular diseases and mortality reduction. When statin use is insufficient for LDL lowering, ezetimibe and PCSK9 inhibitors are recommended as complementary therapies. It is essential to establish lipid-lowering therapy goals based on the stroke subtype and the presence of comorbidities.

## 1. Introduction

Dyslipidemia is a major risk factor for stroke, following hypertension, diabetes, and smoking. Dyslipidemia is also an important risk factor for the prevention and treatment of other vascular diseases such as coronary artery disease and peripheral vascular disease, including stroke [1,2].

The relationship between blood lipids and stroke has been extensively investigated. Total cholesterol (TC) mainly consists of low-density lipoprotein cholesterol (LDL-C) and high-density lipoprotein cholesterol (HDL-C); TC = LDL-C + HDL-C + triglyceride, TG/5. Lipids such as TC, LDL-C, HDL-C, and TG have various effects on pathogenesis of different types of stroke. For example, TC is reported to be inversely correlated to the risk of intracerebral hemorrhage (ICH) with minimal relationship with ischemic stroke. In contrast, high HDL-C was shown to protect against ischemic stroke [2,3,4,5]. Several reports exist regarding TGs and the risk of stroke. TG is also a marker of increased residual cholesterol particles that trigger atherosclerosis and atherothrombosis [2,3,4,5].

LDL-C is the most useful serum lipid marker for predicting the risk of stroke [6]. The risk of ischemic stroke is closely associated with increased LDL-C, and this relationship is especially prominent in the large artery atherosclerosis subtype. Conversely, therapies or medications that lower LDL-C, including statins, decrease the risk of stroke. All recent guidelines describe that LDL-C-lowering therapy should be considered for lifetime to prevent the occurrence or recurrence of stroke, aiming for the “lower is better” approach [7,8,9,10,11,12]. In addition, in meta-analyses of randomized clinical trials, statins were shown to be effective in the prevention of stroke and cardiovascular disease, and regulating LDL-C properly in high-risk or very high-risk patients for cardiovascular disease can reduce the future risk of cardiovascular disease, including stroke [13,14,15]. Although statins were confirmed to significantly lower LDL-C and reduce stroke risk in several studies, the likelihood of cardiovascular diseases remains [3,16,17,18,19]; therefore, to solve this problem, ezetimibe and proprotein convertase subtilisin/kexin type 9 (PCSK9) inhibitors should be considered as an addition to statin treatment.

In this review, the evidence of lipid-lowering medications such as statin, ezetimibe, and PCSK9 inhibitors for secondary stroke prevention and management of dyslipidemia for each subtype of stroke is described.

## 2. Lipid-Lowering Agents for Secondary Stroke Prevention

The characteristics of statin, ezetimibe, PSCK9 inhibitor, and TG lowering agents are described in Table 1.

### 2.1. Statins

**Figure 1:**
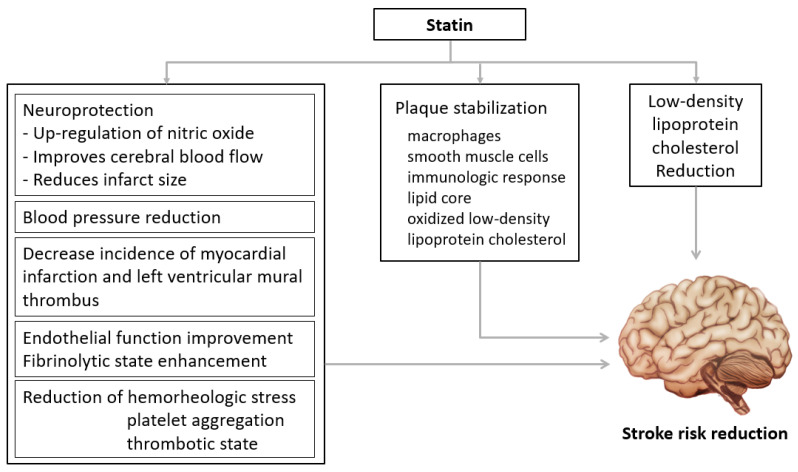
Beneficial effects of statins. Statins have pleiotropic effects such as improvement of vascular endothelial cell function, improvement of nitric oxide bioavailability, antioxidant effects, inhibition of inflammatory response, and stabilization of atherosclerotic plaques.

The advantages of statin treatment were proven in several studies including the Stroke Prevention by Aggressive Reduction in Cholesterol Levels (SPARCL) trial. Statins are the treatment of choice for secondary prevention in stroke patients [16,19,20,21,22,23]. Statins suppress 3-hydroxy-3-methylglutaryl coenzyme A (HMG-CoA) reductase, lower the risk of vascular disease mainly by decreasing LDL-C, and maximum intensity of statins lower LDL-C level by 55–60% from the baseline [8,24,25]. Recent guidelines encourage aggressively lowering LDL-C [7,8,9,10,24,25,26]. In summary, guidelines recommended administration of the maximum tolerable dose of statins primarily and as soon as possible for stroke patients. In addition to LDL-C-reduction effects, statins have several pleiotropic effects such as improvement of endothelial function, upregulation of nitric oxide, antioxidation, suppression of inflammatory response, and stabilization of atheromas (Figure 1) [27,28,29]. These pleiotropic effects are evidence of the usefulness of statins for stroke patients. In a previous study, stroke patients showed good prognosis when taking statins before the onset of the disease [30,31]. Furthermore, statins were also significantly associated with the size of cerebral infarction and the maintenance and development of collateral circulation [30,31].

Among studies regarding statin treatment for acute stroke patients, in the Effects of Very Early Use of Rosuvastatin in Preventing Recurrence of Ischemic Stroke (EUREKA) trial, 20 mg rosuvastatin and placebo were compared in patients who had ischemic stroke within 2 days, and hemorrhagic transformation occurred relatively less in the rosuvastatin group [32]. In an observational study of thrombolysis-treated patients, administration of statins was associated with better future prognosis, especially when administered early or at a high intensity [33]. Furthermore, the risk of hemorrhagic transformation or ICH did not increase despite early or high-intensity statin administration [34,35,36]. Among recent studies, the Treat Stroke to Target (TST) trial is a clinical trial performed in South Korea and France, which confirmed the risk of cardiovascular disease, including stroke, in stroke patients with accompanying atherosclerosis divided into two groups based on LDL-C goals: to have LDL-C reach below 70 mg/dL or LDL-C reach 70–100 mg/dL. In the 70 mg/dL group, the risk of ICH did not significantly increase, and lower risk of cardiovascular disease was observed compared with the 70–100 mg/dL group [35,36,37,38].

Side effects of statins: The notable side effects of statins are diabetes mellitus, muscle symptoms, and hepatotoxicity.

**Figure 2:**
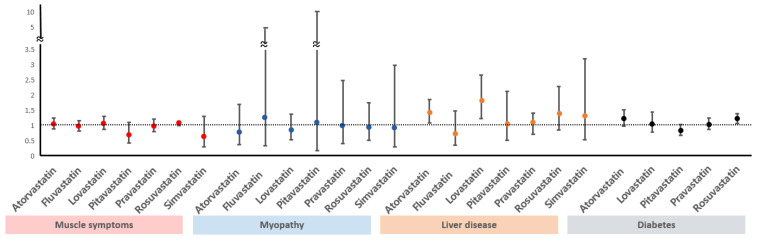
Comparison of side effects among different statins. Horizontal bars indicate ORs with 95% CIs for the complications of statins. Vertical line indicates OR of 1 (no association). ORs, odds ratios; CIs, confidence intervals.

Diabetes mellitus: Statins increase the risk of developing diabetes mellitus dose-dependently, and diabetes mellitus develops mainly with high pre-existing risk of diabetes mellitus such as impaired glucose tolerance [39,40]. Statins may affect plasma glucose levels by weakening insulin resistance, and the mechanism is thought to be the changing of free fatty acid metabolism via HMG-CoA reductase inhibition such as the malfunctioning of pancreatic β cells [41]. Despite these reports, there is no reason to hesitate starting statin therapy due to the risk of diabetes mellitus because the cardiovascular-disease-lowering effect is much greater than the risk of developing diabetes mellitus [24,25,39]. Although some difference in risk of diabetes mellitus among various statins has been reported in several studies [42], further research is needed because this was not proven with a comparison of statin medications in a randomized study of stroke patients. The amount of blood glucose increase differs depending on patient risk factors as well as the statin type and intensity. In a previous study, patients without diabetes mellitus had a blood glucose increase of 3 mg/dL when statins were taken [43]. Atorvastatin and rosuvastatin increased HbA1C by approximately 0.3% in patients who already had diabetes [44]. In a meta-analysis regarding the risk of diabetes mellitus due to each type of statin showed pravastatin had a relatively lower risk than other statins (Figure 2) [45,46,47]. If the clinician or patient is reluctant to use statins due to the risk of diabetes mellitus, lowering statin intensity and using ezetimibe in combination is an alternative. In addition, PCSK9 inhibitors have not been proven to be associated with diabetes development.

**Figure 3:**
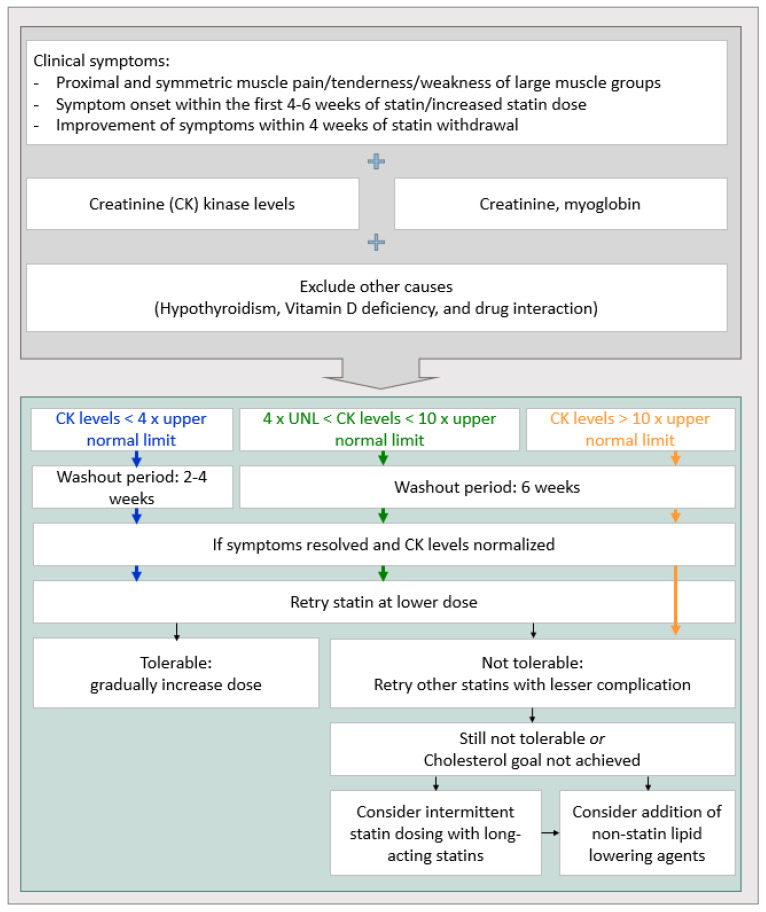
The strategy of statin treatment for muscle toxicity [56].

Muscle-related symptoms: Muscle pain or muscle-related symptoms after administering statins reached 10% in an observational study and approximately 1% in a randomized study [48,49]. Although the likelihood of experiencing muscle-related symptoms is less than 1%, these side effects should not be overlooked due to possible fatal outcomes such as rhabdomyolysis. The creatine kinase (CK) level in blood requires monitoring when the patient complains about muscle symptoms after taking statins [48]. If the CK level is normal or slightly-to-moderately increased (lower than four-fold the normal baseline), whether the cause of muscle symptoms is due to statins needs to be determined. Restarting statin therapy at a low dose or converting to a different statin after 2–4 weeks of statin interruption should be considered. Taking drugs intermittently (for example, once every 2 days or twice a week) is also an option [50]. In stroke patients with very high risk of atherosclerotic cardiovascular disease, if the clinical benefit of statin discontinuation is greater than continuation or if determined safe, statin discontinuation may be considered for over 6 weeks until CK levels are restored [50]. If statin-related muscle symptoms or myopathy are confirmed, discontinuing statin use and a follow-up on CK levels is recommended. Then, changing to another statin or relatively lower-intensity statin can be considered. If the CK level increases up to 10-fold the normal value, interruption of statin use for at least 6 weeks should be considered due to the possibility of statin-induced rhabdomyolysis. If the possibility of rhabdomyolysis is high due to statins, low-intensity and/or ezetimibe combination therapy or a PCSK 9 inhibitor can be considered for patients who require restarting statins, such as patients at high risk of cardiovascular disease (Figure 3). Lastly, statins are not associated with cardiomyopathy [25,50].

**Figure 4:**
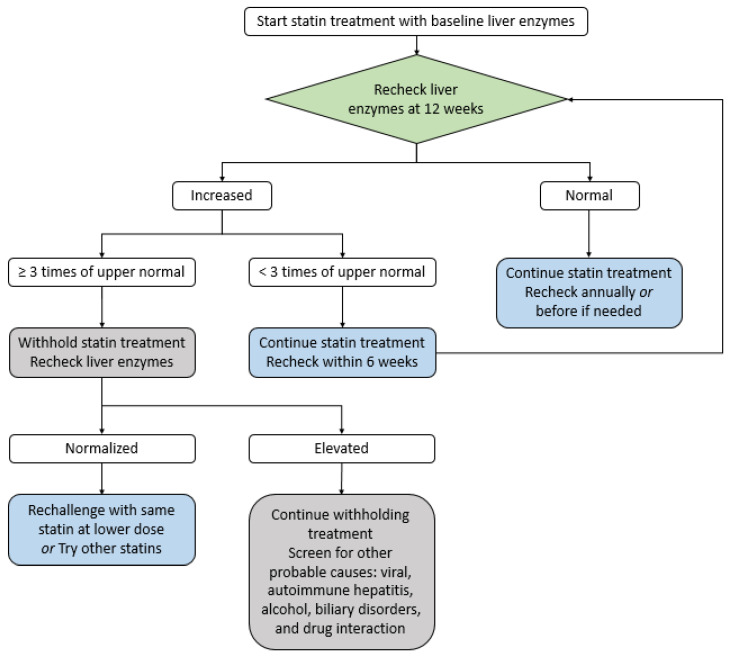
The strategy of statin treatment for liver toxicity [56].

Liver toxicity: Although a slight increase of transaminase occurs in approximately 1% of statin-treated patients, it is not clinically significant and usually normalizes in 3 months without specific treatment [39,51,52]. In general, statin-related severe liver toxicity is very rare. In addition, statins are normally safe for chronic liver disease patients but contraindicated in active liver disease patients [39,51,52]. If there is an increase in liver enzymes less than three-fold the normal value, the statin therapy should be continued, and liver function re-evaluated after six weeks. In cases where liver function exceeds 3-fold the normal value, statins should be discontinued and a re-evaluation performed after 4–6 weeks; if liver function is normalized, a lower-intensity or a different type of statin should be administered. If liver function is consistently deteriorating, other possible causes have to be investigated (Figure 4).

### 2.2. Ezetimibe

Ezetimibe lowers LDL-C by suppressing cholesterol absorption in the gastrointestinal tract [53]. The Improved Reduction of Outcomes: Vytorin Efficacy International Trial (IMPROVE-IT) has proven the benefit of adding ezetimibe to patients undergoing statin therapy. Among recent acute coronary syndrome patients, 10 mg ezetimibe and placebo were compared in patients with LDL-C levels of 50–125 mg/dL despite statin therapy [54]. Administering ezetimibe lowered LDL-C by 16 mg/dL from the baseline resulting in a mean LDC-L level of 54 mg/dL. The ezetimibe group had a 6% lower cardiovascular disease risk, 14% overall stroke risk, and 21% lower ischemic stroke risk without increasing the risk of ICH. A significant difference was not observed in adverse events in both groups. In a following study in which patients with a stroke history in the IMPROVE-IT (n = 641, 3.5%) were analyzed, the ezetimibe group had a 21% lower ischemic stroke risk and no higher risk of hemorrhagic stroke [55]. In the TST trial, most patients received high-intensity statin therapy early after the occurrence of stroke; however, an increased ratio of ezetimibe–statin combination therapy was observed after 1–2 years [37].

As described above, high-intensity statin therapy is recommended primarily for stroke, especially the large artery disease subtype of stroke; however, the LDL-C goal may not be achieved despite high-intensity statin therapy, and stroke recurrence or other atherosclerotic vascular disease such as coronary artery disease is not rare, even if the LDL-C goal has been reached. Furthermore, high-intensity statin therapy accompanies myalgia or myopathy relatively more often and carries a higher risk of diabetes mellitus. Conversely, only 10 mg ezetimibe in a combination therapy showed the same LDL-C-lowering effect as a 2–3-fold increase in statin dose with decreased side effects such as muscle pain with relatively lower diabetes mellitus risk [53]. High-intensity statin therapy is preferably recommended considering its pleiotropic effect; however, ezetimibe–statin combination therapy can also be considered as an alternative. Based on recent lipid management guidelines, maximum tolerated intensity of statin is recommended, and ezetimibe-statin combination therapy can be considered for patients whose LDL-C levels do not reach the goal or patients with very high risk of cardiovascular disease [7,8,9,10,37].

Although several case reports have documented myopathy associated with ezetimibe use [57,58], clinical trials conducted to date have not confirmed an increased risk of myopathy when ezetimibe is used alone or in combination with statins [59,60]. Additionally, while there are case reports discussing its impact on liver function [61], the tendency for ezetimibe to increase liver toxicity, whether used alone or with statins, is known to be similar to that of a placebo [59,60,62,63,64].

In conclusion, considering ezetimibe-statin combination therapy is reasonable for a stroke patient when the desired LDL-C levels have not been achieved. The cardiovascular disease risk is very high even when the LDL-C goal has been met and the patient cannot tolerate optimum intensity due to adverse effects of statin.

### 2.3. PCSK9 Inhibitors

PCSK9 is formed in hepatocytes, released into the bloodstream, and controls the expression of LDL-C receptors by binding with blood LDL-C receptors. Therefore, blood LDL-C increases when PCSK9 is activated, causing the number of LDL-C receptors to decrease. The recently formulated PCSK9 inhibitor suppresses the activation of PCSK9 with monoclonal antibodies and blocks the PCSK9 mechanism, causing the LDL-C receptors to be absorbed and destroyed in hepatocytes, eventually increasing the recycling rate of LDL-C receptors, and lowering LDL-C levels [65].

PCSK9 inhibitors decrease LDL-C by 50–60% from the baseline, lower cardiovascular disease risk similar to statins, and demonstrate strong additive effect when administered in combination with statins [66,67,68]. Thus, PCSK9 inhibitors reduce cardiovascular disease risk alone and in combination with statins despite some cases in actual clinical situations where lowering the LDL-C below the goal is difficult due to the adverse effects of statins or other causes [66,67,68].

The Further Cardiovascular Outcomes Research With PCSK9 Inhibition in Subjects With Elevated Risk (FOURIER) trial proved the effectiveness and safety of PCSK9 inhibitors compared with placebo using the PCSK9 inhibitor evolocumab. The trial was conducted in atherosclerotic cardiovascular disease patients (including 19% ischemic stroke patients) with LDL-C exceeding 70 mg/dL [69]. Compared with placebo, evolocumab lowered LDL-C by 59% from the baseline and maintained it at 30 mg/dL until 48 weeks after administration. Furthermore, 42% of evolocumab-administered patients reached LDL-C of 25 mg/dL. Evolocumab decreased cardiovascular disease risk by 20%, overall stroke risk by 21%, and ischemic stroke risk by 25% compared with the placebo group; however, the risk of ICH was not increased, and adverse effects were similar among treatment groups. In particular, the risk of ICH did not significantly increase even in patients with LDL-C below 25 mg/dL. Furthermore, additional neurological side effects, such as cognitive function, were not significantly different compared with the placebo group [69].

Evaluation of Cardiovascular Outcomes After an Acute Coronary Syndrome During Treatment With Alirocumab (ODYSSEY OUTCOMES) trial results confirmed the effectiveness and safety of PCSK9 inhibitors compared with placebo using alirocumab, another PCSK9 inhibitor. The trial was conducted in patients with acute coronary syndrome (including 3.2% of stroke patients) with LDL-C exceeding 70 mg/dL despite the administration of the maximum tolerated intensity of statins [70]. In the trial, alirocumab reduced LDL-C 55% from baseline and LDL-C level reached a mean value of 53 mg/dL during treatment. The risk of composite cardiovascular events decreased by 15% and ischemic stroke risk by 27% without an increase of hemorrhagic stroke risk in the alirocumab group. Adverse effects were similar among treatment groups [70].

In the ODYSSEY LONG TERM trial, adverse events observed more frequently in the alirocumab group were injection-site reactions, myalgia, neurocognitive events, and ophthalmologic events [71]. Furthermore, the incidence of neurocognitive events and new-onset diabetes mellitus did not differ between patients receiving a PCSK9 inhibitor and those receiving a placebo [69,70]. Clinical trials and meta-analyses have shown that treatment with PCSK9 inhibitors does not increase the risk of incident diabetes mellitus [72,73,74]. A secondary analysis of the FOURIER trial offered insights into the impact of extremely low LDL-C concentrations resulting from PCSK9 inhibition. This analysis revealed that both serious adverse events and adverse events leading to drug discontinuation did not occur more frequently in patients with plasma LDL-C levels below 15 mg/dL [75].

Therefore, considering PCSK9 inhibitors for a stroke patient can be another option if the LDL-C goal is not reached or optimum intensity of statins cannot be administered due to side effects.

### 2.4. Triglyceride-Lowering Agents

Hypertriglyceridemia, along with high LDL, is a recognized risk factor for stroke that has been studied extensively. Several prospective cohort studies and meta-analyses have shown that the likelihood of stroke increases as triglyceride levels rise, particularly in patients with increased waist circumference or metabolic syndrome [76,77,78,79,80]. Although the relative increase in risk is not as significant as with LDL, there is evidence to suggest that patients with hypertriglyceridemia may still benefit from additional interventions beyond statin therapy [76]. Notably, the Veterans Affairs HDL Intervention Trial confirmed the risk of stroke in patients with cardiovascular disease and demonstrated that gemfibrozil, a fibrate medication, reduced the relative risk of stroke by 31% compared to the control group [81]. In the observational studies, low plasma proportion of omega 3-polyunsaturated fatty acids were associated with cerebral small vessel disease and poor outcome after ischemic stroke [82,83]. In the Reduction of Cardiovascular Events with Icosapent Ethyl–Intervention trial (REDUCE-IT), a multicenter randomized controlled trial involving 8179 patients, showed that icosapent ethyl, another triglyceride-lowering agent, significantly reduced the risk of stroke in patients taking statins (hazard ratio 0.75, 95% confidence interval 0.68–0.83; *p* < 0.0001) [84]. On the other hand, the incidence of atrial fibrillation was higher in the icosapent ethyl group compared to the placebo group (5.3% vs. 3.9%), and the rate of peripheral edema was also higher (6.5% vs. 5.0%) [84]. Side effects of fibrates include an increased risk of myopathy, cholelithiasis, and venous thrombosis [85]. Based on these findings, fibrates or icosapent ethyl may be considered as adjunct therapy to statins for patients with hypertriglyceridemia, but further research targeting stroke patients is necessary to establish definitive evidence for secondary prevention.

## 3. Treatment for Stroke Subtypes and Dyslipidemia

Among all types of strokes, 87% of strokes are ischemic, 10% are ICH or hemorrhagic, and 3% are subarachnoid hemorrhagic [1,2]. In the following paragraph, the treatment of dyslipidemia for each stroke subtype will be described.

### 3.1. Large Artery Atherosclerosis

For the large artery atherosclerosis stroke subtype, high-intensity statin therapy is recommended for all ages including patients older than 75 years of age [7,8,9,10,24,25,26,86]. The SPARCL trial showed 80 mg atorvastatin reduced the recurrence risk of all strokes (ischemic + hemorrhagic) up to 42%, especially in the large artery atherosclerosis stroke subtype, compared with placebo, indicating a new paradigm for statin therapy [87]. Hence, high-intensity statin therapy is primarily recommended for stroke patients with accompanying large artery disease. LDL-C usually reaches a specific level approximately 4 weeks after statin administration; however, if the LDL-C goal is not reached despite high-intensity therapy over 4 weeks or high-intensity statin therapy is intolerable due to problems such as adverse effects, additional drug administration including ezetimibe or PCSK9 inhibitors should be considered [7,8,9,10,24,25,26,86]. The guidelines recommend 70 mg/dL as an LDL-C goal for the large artery atherosclerosis stroke subtype; however, the LDL-C goal should be personalized for each stroke patient’s situation because European guidelines have recently suggested a goal of less than 55 mg/dL if patients with the large artery atherosclerosis stroke subtype experience a recurrence of cardiovascular disease or patients have a high recurrence risk. Furthermore, in extreme high-risk groups, the target LDL-C goal of less than 40 mg/dL is recommended. In this subtype of stroke, prolonged dual antiplatelet therapy is often required. Although there is a possibility of hemorrhagic stroke during using dual antiplatelets and high-intensity statins, achieving target LDL level would be effective in preventing secondary stroke and other cardiovascular events. As ezetimibe and PCSK9 inhibitor did not show increased risk of hemorrhagic stroke in IMPROVE-IT, FOURIER trial, and meta-analyses, these drugs may be considered if the risk of intracerebral hemorrhage is high instead of high dose statins [54,69,88].

### 3.2. Small-Vessel Occlusion

Small-vessel occlusion stroke subtype occurs mainly due to hypertension, diabetes, and old age. In addition, atherosclerosis can cause small-vessel occlusion, and atherosclerosis, and the small-vessel occlusion stroke subtype has been shown to be closely related in several studies [89]. In the SPARCL trial, 80 mg atorvastatin lowered the risk of recurrent ischemic stroke by 24% (95% confidence interval, CI, 0.57–1.02) and overall stroke risk by 16% (95% CI, 0.64–1.11) but increased the risk of ICH compared with placebo [90]. Conversely, sub-analysis results in the TST trial conducted in South Korea and France did not show significantly different outcome variables due to the small-vessel occlusion stroke subtype, especially hemorrhagic stroke occurrence, between the group with LDL-C goal of 70–100 mg/dL and the group with LDL-C goal of less than 70 mg/dL [37]. Therefore, in principle, the small-vessel occlusion stroke subtype can be treated similar to the large artery atherosclerosis stroke subtype; high-intensity statin therapy should be initially administered for small-vessel occlusion stroke subtype, and ezetimibe or PCSK9 inhibitors can be considered if LDL-C exceeds 70 mg/dL at follow-up.

In the authors’ opinion, LDL-C should be maintained below 70 mg/dL as a general rule in the small-vessel occlusion stroke subtype, but the value should be constantly monitored and compared with large artery atherosclerosis stroke subtype. In particular, when a small-vessel occlusion stroke subtype patient has a history of ICH or several microbleeds on brain imaging, whether to maintain long-term administration of high-intensity statin therapy should be determined considering both benefit and harm even when pleiotropic effects of statins are taken into account.

### 3.3. Cardioembolic Stroke

Evidence regarding lipid-lowering agents, especially statins, in cardioembolic stroke is scarce, even in randomized controlled trials. The benefit of statins was not proven in the SPARCL trial because patients with atrial fibrillation and other causes of cardioembolism were excluded [87]. In a recent report, lipid-lowering therapy showed benefits in atrial fibrillation patients, similar to the large artery disease and small-vessel disease stroke subtypes [91]. The increased LDL-C in atrial fibrillation was an independent predictor of stroke risk in previous research [92]. Statins reduced LDL-C and exerted pleiotropic effects that enabled statins to decrease stroke risk apart from their lipid-lowering effects [29]. Furthermore, statins lower the likelihood of developing atrial fibrillation that occurs after a previous episode and recurrent atrial fibrillation [93,94,95]. In research conducted on stroke patients with atrial fibrillation, high-intensity statin therapy showed lower risk of composite cardiovascular events than low-to-medium-intensity statin therapy [96].

Limited RCT data exists on the potential risk of hemorrhagic stroke with the use of high-intensity statins in cardioembolic stroke. The Korean nationwide ATrial fibrillaTion EvaluatioN regisTry in Ischemic strOke patieNts (K-ATTENTION) study, which focused on acute ischemic stroke patients with atrial fibrillation, found that the use of high-intensity statins was associated with a lower risk of three-year mortality from any cause, stroke, acute coronary syndrome, or major bleeding compared to low-to-moderate statin use (hazard ratio 0.76; 95% CI 0.59–0.96), with no significant difference in major bleeding between the two groups [96]. A meta-analysis of observational studies also did not show an increased risk of major bleeding with statin use in cardioembolic stroke [86,97]. Although current evidence on statin intensity and administration lacks randomized control trial data, several studies suggest that statin use does not increase hemorrhagic transformation, and given the accompanying atherosclerotic burden, statin administration appears to be a viable option. If multiple cerebral microbleeds or previous intracranial hemorrhage history are accompanied, moderate-intensity statin or ezetimibe can be another option rather than high-intensity statin.

Therefore, treatment of dyslipidemia should not be delayed even in the cardioembolic stroke subtype. In the authors’ opinion, administering more than moderate-intensity statin therapy and adjusting LDL-C to at least less than 70 mg/dL after an acute stroke is recommended, even if a standard for statin intensity or LDL-C has not been established. In addition, prescribing statins is particularly necessary in cases of accompanying atherosclerotic cardiovascular diseases.

### 3.4. Stroke of Undetermined Cause

In the SPARCL trial, the 80 mg atorvastatin group had 20% lower risk of overall stroke (ischemic + hemorrhagic; 95% CI, 0.62–1.27) than the placebo group among ischemic stroke patients with unknown or other causes [90]. One-fourth of stroke cases have unclear origin but close monitoring has shown most were due to large artery disease or paroxysmal atrial fibrillation [98,99]. Although further clinical trials are needed, high-intensity statin therapy should therefore be considered even when the mechanism of stroke is uncertain and there are no contraindications. In particular, high-intensity statin therapy should be considered in cases of accompanying atherosclerotic cardiovascular diseases.

### 3.5. Transient Ischemic Attack

Although randomized research regarding statin administration only for transient ischemic attack patients is lacking, the 80 mg atorvastatin group had 20% lower overall stroke risk (95% CI, 0.60–1.24) than the placebo group among transient ischemic attack patients registered in the SPARCL trial [90]. Together with previous study results, starting high-intensity statin therapy and setting the target LDL level preferably to less than 70 mg/dL for patients at high risk of developing stroke in 1–5 years after transient ischemic attack is reasonable [100,101].

### 3.6. ICH

Data regarding lipid-lowering therapy for patients with ICH history are scarce. Among 4731 patients included in the SPARCL trial, only 93 had a history of ICH [87]; ICH occurred in 88 patients and was associated with older age, cerebral hemorrhage history, poorly controlled hypertension, and the administration of 80 mg atorvastatin. Whether 80 mg atorvastatin has any benefit for stroke patients with a history of ICH is difficult to determine due to the small sample size of 88 subjects and that information regarding ICH location (lobar or non-lobar) was insufficient [87,90,102].

However, the safety concern associated with 80 mg atorvastatin administration in patients with ICH has been suggested in several studies. Issues regarding the administration of 80 mg atorvastatin in patients with ICH include the antithrombotic effect of statins and the relationship between high-intensity statin therapy and the severity of microbleeds, especially the burden of lobar microbleeds, have been reported. In addition, in randomized clinical trials for secondary prevention after ischemic stroke, including the SPARCL trial, statins tended to be associated with future risk of ICH [87,103,104,105]. In summary, determining whether statin administration, especially high-intensity statin therapy, is necessary or appropriate in patients with a history of ICH who are at risk of future ischemic cardiovascular disease, taking into account the effectiveness and side effects, is important. In the decision model announced in 2011 after the SPARCL trial, statin therapy was avoided in cases of lobar ICH if not previously taken, and in cases of deep ICH, statin therapy was recommended only in the extremely high-risk group of cardiovascular disease [106].

Recent research results indicate considering statin administration after the occurrence of ICH. First, the recurrence risk of cardiovascular disease, including death, decreased when continuing statin therapy after ICH; however, the risk of poor prognosis was increased when statin therapy was discontinued after ICH [107,108]. Second, statin therapy can be considered for patients with high risk of ischemic cardiovascular disease even if they have a history of ICH because the recurrence risk of ICH is much lower than the risk of developing ischemic stroke or ischemic cardiovascular disease (deep ICH 1.5–2%/year, lobar ICH 6–7%/year), and the likelihood of developing ICH decreases significantly 1 year after the occurrence of ICH [109,110,111,112]. Three, statin administration is likely to be of great benefit, especially in cases of deep ICH, because future risk of ischemic cardiovascular disease is higher than ICH risk; however, the needs of each patient should be taken into consideration because choosing statin intensity or dose has yet to be established [109,110,111,112].

Therefore, for ICH patients who have previously taken statins and are at risk of ischemic cardiovascular diseases, continuing statin therapy is reasonable. For patients who have not previously taken statins, considering statin treatment in cases of deep ICH is reasonable if no contraindication exists because the future risk of cardiovascular disease is higher than of ICH; however, caution is recommended when starting statin therapy in cases of lobar ICH.

## 4. Concomitant Risk Factors and Their Effect on Lipid-Lowering Agents

### 4.1. Hypertension

High blood pressure is a common risk factor in stroke patients, and it increases the risk of developing atherosclerotic cardiovascular disease [113,114,115]. The dyslipidemia treatment guideline categorizes hypertension as a risk factor and recommends the use of statins if indicated [7,19]. Currently, there is no evidence to suggest a different statin treatment goal for stroke patients with hypertension. Hypertensive patients often experience dyslipidemia, and since these two conditions are mechanistically related, they should be managed together. According to the Anglo-Scandinavian Cardiac Outcomes Trial-Lipid Lowering Arm (ASCOT-LLA) study [116], the statin-combined group had a lower incidence of stroke and coronary events (hazard ratio 0.64 [95% CI 0.50–0.83], *p* = 0.0005) during the 3.3-year follow-up period. Therefore, it is important to screen for dyslipidemia and actively treat stroke patients with hypertension and dyslipidemia; however, further research is necessary to determine which statins are most effective in treating patients with stroke and hypertension, as there is currently a lack of trials on this subject.

### 4.2. Diabetes Mellitus

Although statin use is associated with new-onset diabetes mellitus, it is strongly recommended for diabetic patients [40]. Multiple studies have demonstrated that using statins in diabetic patients can reduce the incidence of cardiovascular events. In a randomized placebo-controlled trial of simvastatin (MRC/BHF Heart Protection Study), 5963 diabetic patients experienced a 22% decrease in major vascular events, including stroke [117]. Similarly, the atorvastatin group in the Collaborative Atorvastatin Diabetes (CARDS) Study exhibited a reduction of 38% in major vascular events [118]. In the IMPROVE-IT trial, when ezetimibe was used in combination with simvastatin, the primary composite endpoint decreased by 15% in diabetic patients compared to simvastatin alone (HR 0.85, 95% CI 0.78–0.94) [54,119]. In the FOURIER trial, a subgroup of 11,031 diabetic patients showed a 17% decrease in the primary endpoint with the use of additional PSCK9 inhibitors compared to the statin group (HR 0.83, 95% CI 0.75–0.93; *p* = 0.0008) [120]. Additionally, in the renal effects of atorvastatin and rosuvastatin in patients with diabetes who have progressive renal disease (PLANET) I trial, which investigated the use of statins in diabetic patients with proteinuria, high-dose atorvastatin demonstrated a renoprotective effect, reducing the urine protein:creatinine ratio compared to rosuvastatin [121]. The ADA and ACC/AHA guidelines recommend the use of moderate-intensity statins for diabetic patients aged 40–75 with an LDL goal of less than 100 mg/dL and an LDL goal of less than 70 mg/dL if a cardiovascular risk factor is present for primary prevention [19,122]. For secondary prevention of stroke, controlling LDL levels in diabetic patients is essential. Although there are no statins unsuitable for patients with diabetes mellitus, using very high-intensity rosuvastatin in those with proteinuria and diabetes mellitus may increase the risk of acute renal failure [123].

### 4.3. Chronic Kidney Disease

In the Kidney Disease: Improving Global Outcomes (KDIGO) guideline, the use of statins in chronic kidney disease (CKD) patients aged 50 years or older is recommended, with the exception of dialysis patients [124]. For CKD patients between the ages of 18 and 49 years, statins are recommended if they have one or more risk factors, such as a coronary disease, diabetes mellitus, ischemic stroke, or 10% or more risk or 10-year incidence of coronary death or nonfatal myocardial infarction. In the KDIGO guideline, moderate doses of statins such as 20 mg atorvastatin, 10 mg rosuvastatin, 40 mg simvastatin, 40 mg pravastatin, 80 mg fluvastatin, or 2 mg pitavastatin are recommended because of a higher possibility of drug side effects due to decreased excretion. Meanwhile, the 2019 ESC/EAS and AHA/ACC guidelines classified patients with an eGFR of <60 mL/min/1.73 m^2^ as a high-risk group and recommended the use of high-intensity statins [7,19]. Moreover, in the Treating to New Targets (TNT) trial, high-dose atorvastatin at 80 mg reduced the first major cardiovascular event by 32% in 3107 CKD patients compared to low-dose atorvastatin [125]. Since decreased renal function is also related to intracranial hemorrhage and cerebral microbleeds [126], it seems necessary to consider the type and intensity of statin in case by case, considering the results of studies such as the SPARCLE trial. It is not recommended to newly start statins in patients on hemodialysis, but they should not be discontinued in patients who are on statins [124].

## 5. Limitation

First, this paper is a narrative review and did not conduct a systematic review or meta-analysis. Second, although authors’ opinion was presented based on current practices or evidence, future updates are needed. Third, since our review is focused on the secondary prevention of stroke, it is difficult to be applied to other diseases, such as cardiovascular disease.

## 6. Conclusions

**Figure 5:**
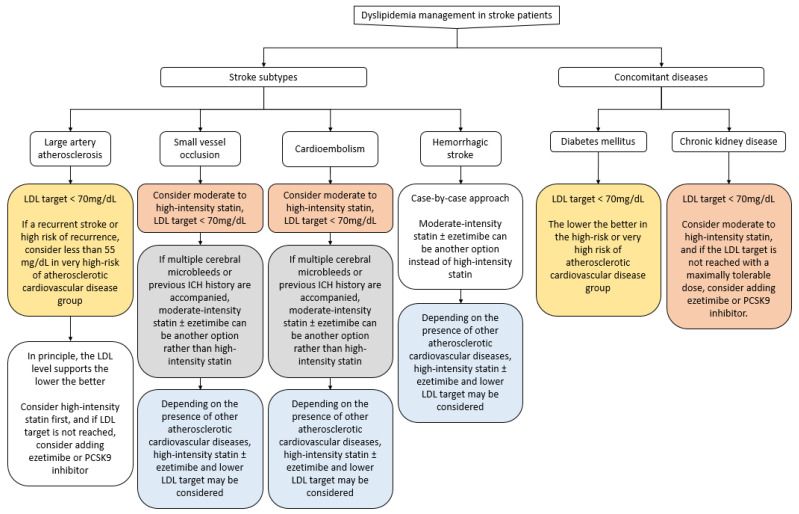
Management of dyslipidemia for secondary stroke prevention.

Statins, ezetimibe, and PCSK9 inhibitors should be administered to stroke patients for secondary prevention. When prescribing medications for dyslipidemia, the combination, types, and dosing have to be carefully chosen based on the patient’s comorbidities and stroke subtypes. Especially for statins, intensity of administration as well as side effects, comorbidities, and patient personal characteristics should be considered. If the LDL-C goal is not achieved or administering the optimum statin dose is difficult due to side effects, ezetimibe or a PCSK9 inhibitor can be considered (Figure 5). Lastly, clinicians should be aware that suspension of statin treatment can cause long-term harm to stroke patients.

## Figures and Tables

**Table 1:** Characteristics of lipid-lowering agents for prevention of stroke.

	Mechanism	Effects	Usage/Dosage	Side Effects
Statins	Inhibit 3-hydroxy-3-methylglutaryl coenzyme A reductase	Inhibition of cholesterol biosynthesis LDL-C-lowering effect 24–52%	Oral, Maximum tolerated dose	Myalgia, myopathy, new-onset diabetes mellitus, elevated liver enzyme
Ezetimibe	Block Niemann-Pick C1-Like 1 in intestine	Inhibition of cholesterol absorption by small intestine LDL-C-lowering effect 19% alone, 21–29% in combination with statins	Oral, 10 mg daily	Myalgia
PCSK9 inhibitors	Inhibit proprotein convertase subtilisin/kexin type 9	Inhibition of LDL recycle from the blood LDL-C-lowering effect 45–70%	Subcutaneous injection, alirocumab 75 to 150 mg, evolocumab 140 mg in 2 weeks or 420 mg in 1 month	Injection site reaction, myalgia, ophthalmic event
Fibrates	Peroxisome-proliferator activated receptor-alpha agonist	Reduction in synthesis of fatty acid and triglycerides Triglyceride-lowering effect 25–50%	Oral, fenofibrate 160 to 200 mg daily, gemfibrozil 600 to 1200 mg daily	Myopathy, cholelithiasis, venous thrombosis
Icosapent ethyl	Multiple mechanisms	Reduction in production of triglycerides, enhancement in clearance of triglycerides Triglyceride-lowering effect 26–45%	Oral, 2–4 g PO q12hr	Myalgia, atrial fibrillation, peripheral edema

LDL-C: low-density lipoprotein cholesterol

## Data Availability

Not applicable.
